# Combination of Low Calcium with Y-27632 Rock Inhibitor Increases the Proliferative Capacity, Expansion Potential and Lifespan of Primary Human Keratinocytes while Retaining Their Capacity to Differentiate into Stratified Epidermis in a 3D Skin Model

**DOI:** 10.1371/journal.pone.0123651

**Published:** 2015-04-13

**Authors:** Xanthe L. Strudwick, Debbie L. Lang, Louise E. Smith, Allison J. Cowin

**Affiliations:** Regenerative Medicine, Mawson Institute, University of South Australia, Mawson Lakes, Australia; University Hospital Hamburg-Eppendorf, GERMANY

## Abstract

Human keratinocytes are difficult to isolate and have a limited lifespan. Traditionally, immortalised keratinocyte cell lines are used *in vitro* due to their ability to bypass senescence and survive indefinitely. However these cells do not fully retain their ability to differentiate *in vitro* and they are unable to form a normal stratum corneum in organotypic culture. Here we aimed to generate a pool of phenotypically similar keratinocytes from human donors that could be used in monolayer culture, without a fibroblast feeder layer, and in 3D human skin equivalent models. Primary human neonatal epidermal keratinocytes (HEKn) were cultured in low calcium, (0.07mM) media, +/-10μM Y-27632 ROCK inhibitor (HEKn-CaY). mRNA and protein was extracted and expression of differentiation markers Keratin 14 (K14), Keratin 10 (K10) and Involucrin (Inv) assessed by qRT-PCR and Western blotting. The differentiation potential of the HEKn-CaY cultures was assessed by increasing calcium levels and removing the Y-27632 for 72hrs prior to assessment of K14, K10 and Inv. The ability of the HEKn-CaY, to form a stratified epithelium was assessed using a human skin equivalent (HSE) model in the absence of Y-27632. Increased proliferative capacity, expansion potential and lifespan of HEKn was observed with the combination of low calcium and 10μM ROCK inhibitor Y-27632. The removal of Y-27632 and the addition of high calcium to induce differentiation allowed the cells to behave as primary keratinocytes even after extended serial passaging. Prolonged lifespan HEK-CaYs were capable of forming an organised stratified epidermis in 3D HSE cultures, demonstrating their ability to fully stratify and retain their original, primary characteristics. In conclusion, the use of 0.07mM Calcium and 10μM Y-27632 in HEKn monocultures provides the opportunity to culture primary human keratinocytes without a cell feeder layer for extended periods of culture whilst retaining their ability to differentiate and form a stratified epithelium.

## Introduction

**Fig 1:**
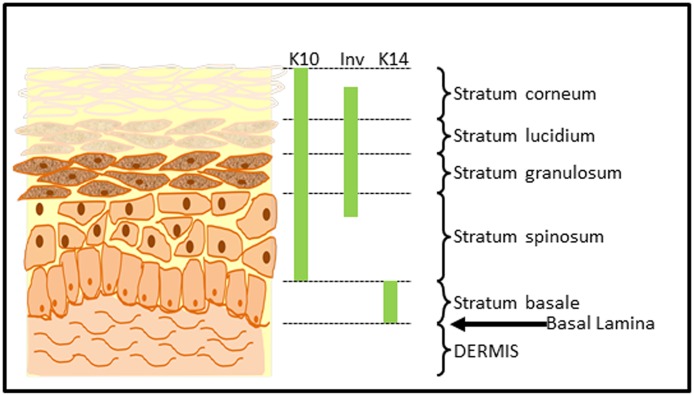
Diagram of human skin showing differentiated keratinocytes of the epidermis and expression of stratification markers Keratin 10 (K10), Keratin 14 (K14) and Involucrin (Inv).

Human skin cells, especially keratinocytes, are difficult to isolate and have a limited lifespan in which they may be used [1]. This often means that repeated isolations of cells are required from multiple donors which not only can be difficult to source and time consuming, but can give rise to experimental variation due to genetic differences, location of skin, patient age and sex. Keratinocytes rapidly differentiate in culture, leading to both a reduction in cell numbers due to the loss of the proliferative, basal cells and also phenotypic variance of cells in culture, over time [2,3]. These confounding factors greatly affect the reproducibility and accuracy of *in vitro* keratinocyte experiments. In addition it is well known that keratinocytes *in vivo* undergo a process of differentiation, forming a multi-layered epidermis and stratum corneum to form a functional skin barrier (Fig 1). In vitro human skin explant models have been developed which allow investigations concerning keratinocyte differentiation and skin barrier formation to be assessed however these models require the effective maintenance of a basal pool of keratinocytes with full differentiation capabilities.

Traditionally, immortalised keratinocyte cell lines have been used *in vitro* due to their ability to bypass senescence and survive indefinitely in culture. The most commonly used human keratinocyte cell line, HaCaT’s, was spontaneously immortalised from adult human keratinocytes [4]. The HaCaT cell line, cannot however, be considered analogous to the primary phenotype of keratinocytes, as it has acquired a p53 UV-linked genetic mutation [5]. In fact, the cell line has been described as representative of epithelial cells in the early stages of carcinogensis [6–8] and investigations of the HaCaT cell line during long term culture have revealed some phenotypic variations which can differ greatly from the original phenotype, and also between laboratories and over time [9]. Contrary to original belief, these cells do not fully retain the ability to differentiate *in vitro* and they are unable to form a normal stratum corneum in organotypic culture [10]. It has often therefore been necessary to return to primary cell culture with all its limitation and difficulties in order to investigate how keratinocytes respond under different conditions.

The rho kinase (ROCK) inhibitor Y-27632 has been shown to inhibit terminal differentiation and increase proliferation in keratinocytes in culture [11]. Primary keratinocytes co-cultured with irradiated fibroblast feeder cells, in keratinocyte growth media supplemented with 10μM Y-27632, maintain proliferation for up to 190 population doublings (PD) and as such it has been suggested that they be considered immortal [12,13]. Unlike HaCaT cells, the karyotype of Y-27632 immortalized cells remains normal and the morphology of the cells continue to resemble that of basal keratinocytes. Where the HaCaT cell line exhibits a p53 UV-linked mutation, Y-27632 treated keratinocytes continue to express p53 and exhibit a normal DNA damage response. Moreover, the removal of Y-27632 from the culture media allows these cells to maintain a primary phenotype, forming a stratified epithelium in a 3D model of keratinocyte differentiation [12]. However these keratinocytes could not be sustained in the absence of a feeder layer under these conditions, with cultures undergoing senescence after around 35 population doublings [12].

Changes in the concentration of calcium have been shown to affect keratinocyte differentiation. High levels of calcium stimulate differentiation of keratinocytes while low calcium has been used to prolong the lifespan of basal primary keratinocytes in culture by preventing loss of basal cells due to keratinocyte differentiation [14]. Keratinocyte growth media has been formulated commercially with less than 0.1mM calcium to promote the maintenance of a basal proliferative keratinocyte population.

The purpose of this study was to generate a pool of phenotypically similar keratinocytes from human donors that could be used in both monolayer culture and 3D human skin equivalent models without a cell feeder layer. Using freshly isolated human keratinocytes we investigated using very low calcium in combination with ROCK inhibition to prolong the lifespan of keratinocytes in culture whilst maintaining the primary keratinocyte phenotype. Assessments of proliferative capacity, expansion potential and lifespan of primary human keratinocytes and the retention of the capacity to differentiate into stratified epidermis in a 3D skin model were performed.

## Methods

### Human Keratinocyte Culture

Primary human epidermal keratinocytes (HEKn) were isolated from donor neonatal foreskin, collected under human research ethics approval from the Calvary Health Care Adelaide Human Research Ethics Committee (11-CHREC-F007), in accordance with the Declaration of Helsinski principles. Written informed consent was obtained from the next of kin, caretakers, or guardians on behalf of the children enrolled in the study. The written consent form was approved by the human research ethics committee which was signed prior to the procedure occurring.

The epidermis was separated from the dermis via overnight incubation with a 10mg/ml Dispase (Sigma, St Louis, Missouri) solution containing 100 IU/ml penicillin, 100μg/ml streptomycin and 0.25μg/ml amphotericin B (Sigma, St Louis, Missouri), followed by TrypLE (Invitrogen, USA) dissociation of the keratinocytes within the epidermal sheet. Cells were then centrifuged at low speed (160 x g for 5 min) and seeded for culture in CELLnTEC (Bern, Switzerland) low calcium, (0.07mM) ‘CnT-07’ media containing 100 IU/ml penicillin, 100μg/ml streptomycin and 0.25μg/ml amphotericin B (Sigma St Louis, Missouri) at a density of 5 x 10^4^ cell/cm^2^ in standard tissue culture vessels.

The cells were grown in low calcium media with or without supplemental 10μM Y-27632 Rock Inhibitor (Sigma, St Louis, Missouri) which was added at the time of initial seeding. These cells are abbreviated as HEKn-CaY. Following the first 2 passages, performed when the cells reached 90% confluence, antibiotics and antimycotics were removed from the culture media and population doubling (PD) rates assessed during serial culture. Cell number and viability were calculated using trypan blue exclusion during passage at 90% confluence. Population doubling was calculated using the equation: n = 3.32 (log UCY—log I) + X, where n = the final PD number at the end of a given subculture, UCY = the cell yield at that point, I = the cell number used as inoculum to begin that subculture, and X = the doubling level of the inoculum used to initiate the given subculture [15].

HaCaT keratinocytes (Cell Lines Service, DKFZ, Heidelberg, Germany) were cultured under routine conditions in Dulbecco’s Modified Eagle Medium [High Glucose, 2mM L-glutamine] (Life Technologies, Mulgrave, Victoria, Australia) supplemented with 10% Fetal Calf Serum.

### mRNA and Protein isolation and assessment

mRNA and protein was extracted from monolayers of HEKn and HaCaT cells using AllPrep Protein/RNA Isolation Kit according to manufacturer’s protocol (QIAGEN GmBH, Hilden Germany), quantified using the NanoDrop Lite Spectrophotometer (ThermoScientific, Wilmington, USA) and expression of differentiation markers Keratin 14 (K14), Keratin 10 (K10) and Involucrin (Inv) assessed by qRT-PCR and Western blot. mRNA expression was quantified as relative normalised expression against multiple reference genes [β-2-microglubulin (B2M) and Tyrosine 3-monooxygenase/tryptophan 5-monooxygenase activation protein (zeta polypeptide) (YWHAZ)] following iScript cDNA synthesis (BioRad, California, USA) and qPCR on the BioRad CFX Connect using SsoAdvanced SYBR Green Universal Supermix synthesis (BioRad, California, USA) with the following PCR primers: K14 Forward 5’-CATGAGTGTGGAAGCCGACAT-3’, K14 Reverse 5’-CATGAGTGTGGAAGCCGACAT-3’; K10 Forward 5’-TTGCTGAACAAAACCGCAAAG-3’, K10 Reverse 5’-GCCAGTTGGGACTGTAGTTCT-3’; Inv Forward 5’-GACTGCTGTAAAGGGACTGCC-3’, Reverse 5’-CATTCCCAGTTGCTCATCTCTC-3’; B2M Forward 5’-GATGAGTATGCCTGCCGTGTG-3’, B2M Reverse 5’-CAATCCAAATGCGGCATCT-3’; YWHAZ Forward 5’-ACTTTTGGTACATTGTGGCTTCAA-3’, YWHAZ Reverse 5’-CCGCCAGGACAAACCAGTAT-3’.

Protein expression was confirmed by standard Western blotting of 50μg protein on nitrocellulose membrane with β-tubulin as loading control. Briefly, blots were incubated overnight at 4°C with primary antibodies for β-tubulin (Clone B-5-1-2, 1:10,000, Sigma-Aldrich, St Louis, MO, USA), K14 (Clone LL002, 1:1000, ThermoScientific, Cheshire, UK), K10 (EP1607IHCY, 1: 25,000, Abcam, Cambridge, UK) and Inv (Clone Sy5, 1:1000, ThermoScientific, Cheshire, UK) after blocking with 5% skim milk powder (Diploma Instant, Mt Waverley, Vic, Australia). Secondary incubations were then performed with HRP-conjugated Goat anti Mouse IgG, 1:2000 for β-tubulin, K14 and Inv, and Goat anti Rabbit IgG, 1:2000 for K10 (both from Dako, Carpinteria, CA, USA) for 1hr at room temperature and bands revealed using SuperSignal West Femto Chemiluminescent Substrate (Pierce, ThermoScientific, Rockland, USA) and visualised using G:Box (Syngene, Cambridge, UK).

### 2D Differentiation Assay

To check for differentiation potential of the late passage HEKn-CaY treated cells, 2D differentiation assays were established, and expression of keratinocyte differentiation markers assessed at the protein level. Passage 10 HEKn-CaY were seeded at 2.5 x 10^3^ cells /cm^2^ in LabTek II chamber slides (Nunc, Rochester, New York) in CnT-07 supplemented with 10μM Y-27632. Cells were cultured 37°C, 5% CO_2_ until 90% confluence achieved, then media changed to CnT-02 2D Differentiation Media, 0.07mM calcium (CELLnTEC, Bern, Switzerland) in the absence of Y-27632 for 24 hrs, followed by a further 72 hrs culture in media raised to 1.2mM calcium. Cells were fixed onto slides with 2% paraformaldehyde and permeabilized with 0.2% Triton X-100 prior to blocking with 3% normal goat serum and detection of stratification markers by immunocytochemistry. Primary antibody incubations were performed for 1hr (K10 Clone DE-K10, K14 Clone LL002 and Inv Clone Sy5; all 1:200, ThermoScientific, Cheshire, UK), followed by detection with species-specific, Alexa-488 conjugated secondary antibodies (1:200, Molecular Probes, Life Technologies, Waltham, USA). A nuclear counterstain was also performed using 4',6-Diamidino-2-Phenylindole, Dihydrochloride (DAPI) at 1:5000 for 2 mins (Sigma-Aldrich, St Louis, MO, USA).

### Human Skin Equivalent Model

To investigate the stratification ability of the cultured keratinocytes, HSEs were prepared using 3x10^5^ HaCaT, passage 10 HEKn-CaY or passage 3 HEKn and 1x10^5^ passage 6 human foreskin fibroblasts on acellular de-epidermised dermis (DED) in Green’s media (1.725μM calcium) in the absence of Y-27632 according to protocol described by McNeil et al [16]. 21 days after raising to air-liquid interface, the HSEs were fixed in 10% buffered formalin and embedded in paraffin wax for histological analysis. 4μm histological sections were stained with haematoxylin and eosin (H&E) and differentiation markers were assessed by immunofluorescence following antigen retrieval according to manufacturer’s protocols (DAKO Glostrup, Denmark) with trypsin digestion. Following blocking in 3% normal goat serum, primary antibodies against K10, K14 and Inv (1:200, ThermoScientific, Cheshire, UK) were applied, followed by detection with species-specific, Alexa-488 conjugated secondary antibodies (1:200 Molecular Probes, Life Technologies, Waltham, USA). For verification of staining non-specific binding was determined by omitting primary or secondary antibodies. All control sections had negligible immunofluorescence.

### Statistical analysis

Statistical differences were determined using the Student’s t-test or an ANOVA. For data not following a normal distribution, the Mann—Whitney U-test was performed. A P-value of less than 0.05 was considered significant.

## Results

### Primary keratinocytes can be maintained in a proliferative basal state in very low calcium and with ROCK inhibition without a cell feeder layer for greater than 40 population doublings

**Fig 2:**
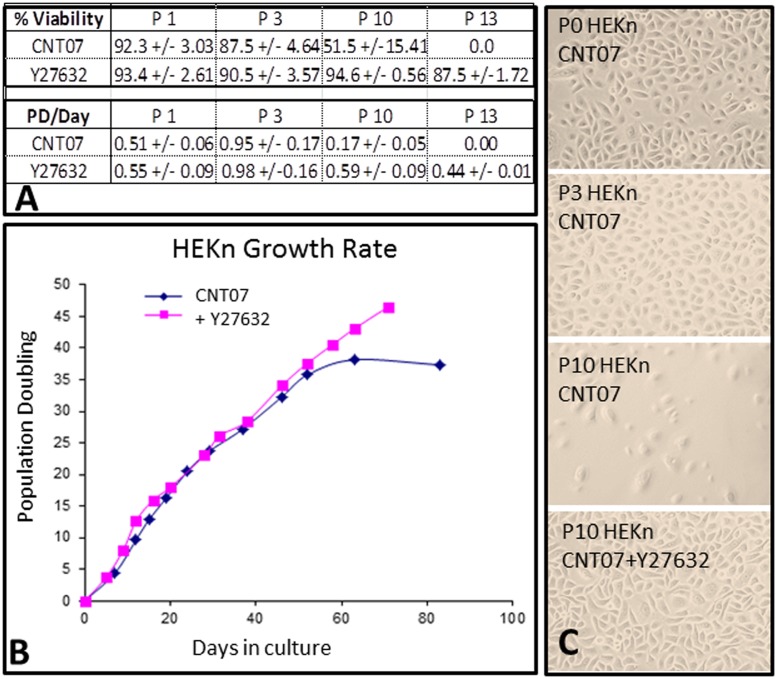
Neonatal human keratinocytes were cultured in CellnTec basal media (containing 0.07mM calcium) or basal media supplemented with 10μM Y-27632. Cells were assessed for cell viability at each passage, performed at 90% confluence. Trypan blue exclusion assay differentiated between viable and non-viable cells based upon membrane permeability and growth rate calculated as population doubling per day (A). Growth rate is represented graphically as population doubling over days in culture (B). Phase contrast images at 20x magnification demonstrate morphology of primary passage 3 and 10 HEKn cultured in CnT-07 alone and passage 3 and 10 HEKn-CaY supplemented with Y-27632 (C). n = 3 isolations from separate donors. Data is expressed as Mean +/- SEM. Images are representative of three experimental repeats.

Human neonatal epidermal keratinocytes (HEKn) cultured in low, 0.07mM calcium basal media (CnT-07) reached senescence, defined as less than 0.2 PD/day within 4 weeks and after 35PD which is in line with expected culture rates described by the manufacturer (CELLnTEC). However, cell viability was low, with trypan blue exclusion assay showing cell viability dropping from 87.5% at the third passage to around 50% after 10 passages and 0% at passage 13 (Fig 2A). This is indicative of the cells undergoing terminal differentiation and the loss of basal keratinocytes from culture [17]. In contrast, HEKn cultured in low, 0.07mM calcium basal media (CnT-07) supplemented with 10μM Y-27632 ROCK inhibitor (HEKn-CaY) maintained a high (95%) viability and a steady growth rate for greater than 40 population doublings (Fig 2B). The addition of 10μM Y-27632 increased proliferation rates with a growth rate of 0.5PD per day being maintained in late passages (beyond 10 passages). At passage 13, around 48 PD, cell viability remained high at an average of 87.5% and the growth rate continued at 0.44PD/day (Fig 2A). Phase contrast microscope images confirm the macroscopic phenotype was similar between the passage 10 HEKn-CaY and that of the primary and passage 3 HEKn usually used in *in vitro* experiments (Fig 2C). In addition to the inability of passage 10 HEKn cells to reach confluence due to the decline in proliferation and low viability of these cells, the cells exhibited marked changes in morphology including increased size and more rounded shape. As such, these cells were not included in further investigations.

### HEKn-CaY basal phenotype is confirmed with expression of basal keratinocyte markers only when cultured with 10μM Y-27632 Rock Inhibitor in 0.07mM Ca^2+^

**Fig 3:**
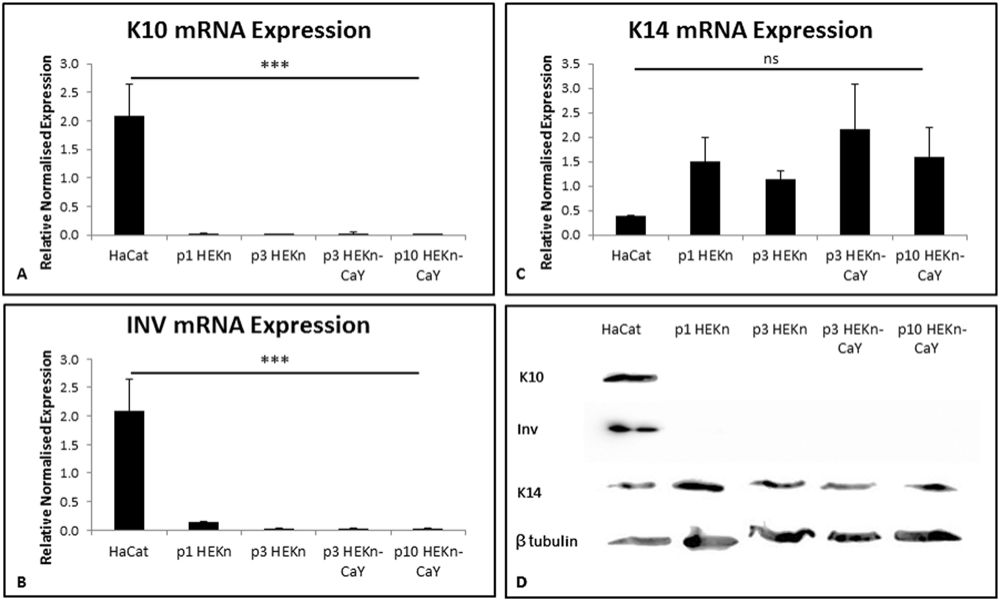
mRNA was extracted from HaCaT, HEKn cells (passage 1 & 3) or HEK-CaY (passage 3 & 10) and expression of differentiation markers K10 (A) and Inv (B) or basal marker K14 (C) assessed by qRT-PCR normalised to multiple reference genes B2M and YWHAZ. Data is expressed as relative normalised expression, Mean +/- SEM, n = 3. *** = p<0.001, ns = not significant, p>0.05, ANOVA. Isolated protein was assessed by western blot to confirm expression with β-tubulin used as a loading control (D). Images are representative of three experimental repeats.

RNA and protein were extracted from monolayer cultures of HaCaT, passage 1 HEKn, passage 3 HEKn, passage 3 HEKn-CaY and passage 10 HEKn-CaY. qRT-PCR and Western blot were performed to assess the expression of differentiation markers K14, K10 and Inv with HaCaT lysate used as controls (Fig 3). K14 is a marker of basal keratinocytes. K10 is an early marker of differentiation and is expressed by keratinocytes in the suprabasal layers of the epidermis. Inv is a terminal differentiation marker and is expressed by cells within the cornified envelope of the cornified layer of the epidermis. No mRNA expression of the differentiation markers K10 (A) or Inv (B) was observed in HEKn or HEKn-CaY cells indicating they neither of these cell populations had undergone differentiation. Expression of K14 mRNA was observed in all cell populations with no significant difference in levels, confirming their basal phenotype (C). Protein expression (D) confirmed the basal phenotype of HEKn-CaY cells with K14 expression detected in both early (passage 3) and late (passage 10) cultures, similar to that observed with both the primary and passage 3 HEKn cultures. The HaCaT cell line showed a more differentiated phenotype with K10 and Inv expression detected at significantly increased levels.

### HEKn-CaY cells express early differentiation marker in 2D differentiation model

**Fig 4:**
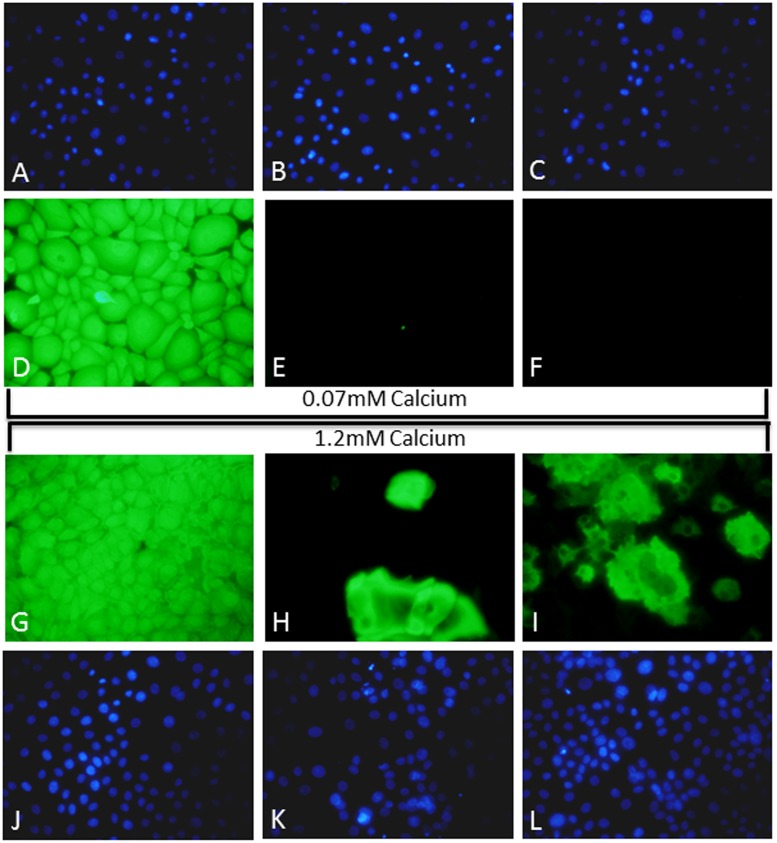
Passage 10 HEK-CaY were seeded onto LabTek II Chamber slides for the 2D differentiation model. Cells were cultured for 72hrs in either 0.07 calcium and assessed for K14 (D), K10 (E) or Inv (F) expression by immunocytochemistry or 1.2mM calcium and K14 (G), K10 (H) or Inv (I) expression assessed. DAPI nuclear counterstain was also performed on cells grown in 0.07mM (A-C) and 1.2mM (J-L) calcium to confirm the presence of live cells. All images are taken at 20x magnification and are representative of three experimental repeats.

The potential of the passage 10 HEKn-CaY cells to differentiate was assessed using a 2D monolayer culture and using high calcium exposure to induced differentiation. Expression of differentiation markers was investigated using immunocytochemistry (Fig 4). K14 expression was observed in both the low level 0.07mM calcium treated keratinocytes (D) and in high 1.2mM calcium induced cells indicating the presence of basal cells (G). K10 expression was absent in the low calcium cultures (E) however it was induced in the high calcium treated cultures (H) indicating that the HEKn-CaY cells had differentiated into different types of keratinocytes. Inv was absent in low calcium treated cells (F) but was also observed in the high calcium treated cells suggesting that some of these cell had undergone terminal differentiation (I). DAPI nuclear counterstain confirms the presence of cells (A-C, J-L) even where no K10 or Inv staining was observed in 0.07mM calcium treated keratinocytes (B,C).

### HEKn-CaY but not HaCaT’s are capable of forming fully stratified epidermis in 3D human skin equivalent culture

**Fig 5:**
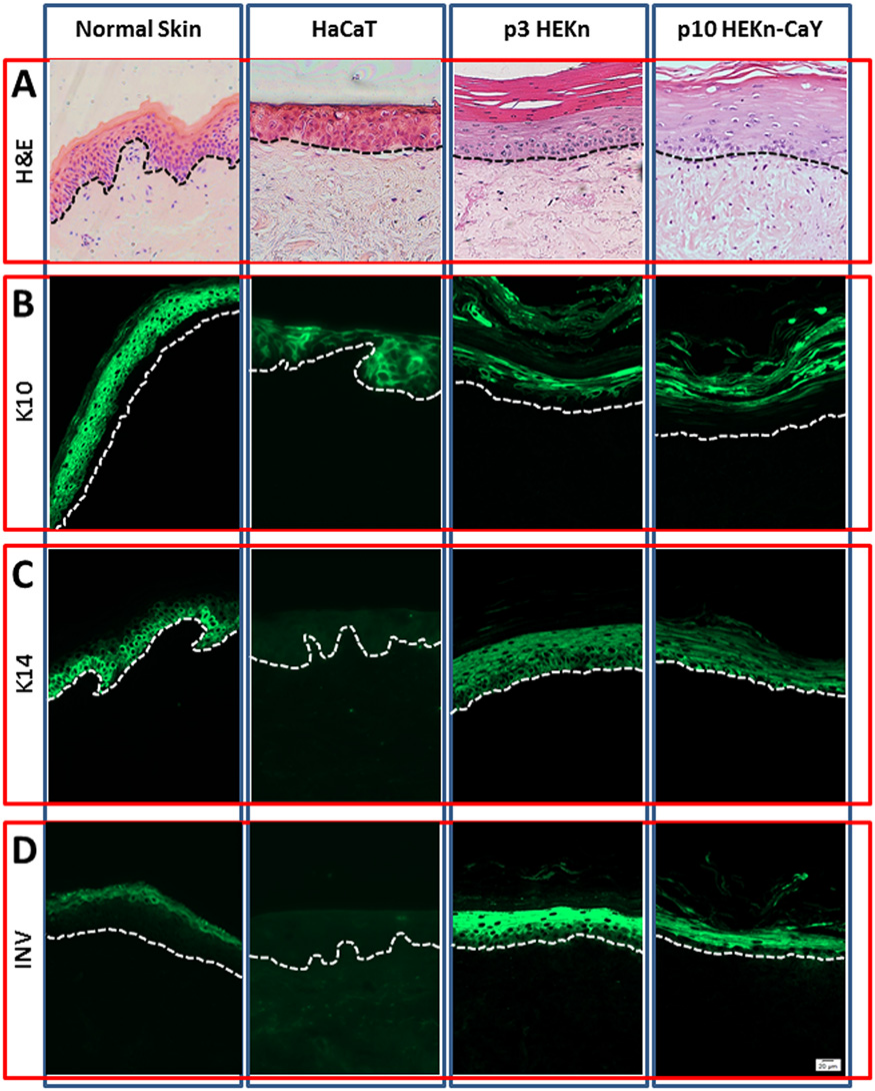
HaCaT, HEKn (passage 3) and HEK-CaY (passage 10) cells were cultured with human foreskin fibroblasts in a 3D human skin equivalent model for 28 days *in vitro*. Gross morphology was compared to normal human skin by H&E (A) to assess ability to for an organised stratified epidermis. Expression of stratification markers K14 (B), K10 (C) and Inv (D) was also assessed by immunohistochemistry. Dashed lines indicate the location of the basement membrane. Scale bar is equivalent to 20μm. All images are taken at 20x magnification and are representative of three experimental repeats.

Passage 10 HEKn-CaY cells and HaCaT’s were stratified in a 3D human skin equivalent (HSE) culture and gross morphology compared to normal human skin and a 3D skin equivalent created with passage 3 HEKn as controls (Fig 5). H&E sections (A) reveal that HaCaT cells form a disorganised epithelium with no observable cornified layer. The gross morphology of both passage 3 HEKn and passage 10 HEKn-CaY appear more similar to normal epithelium complete with evidence of full stratification and cornification. Immunofluorescent images of stratification markers K10 (B), K14 (C) and Inv (D) in green demonstrates that HEKn-CaY but not HaCaT’s are capable of forming fully stratified epidermis in a manner similar to low passage HEKn cells. HSE prepared with HaCaT’s express K10 but not K14 nor Inv suggesting a more suprabasal keratinocyte phenotype that are not capable of stratification to form the cornified epithelium. HEKn and HEKn-CaY cells however both express K14 in the basal cells, K10 in the suprabasal layers and exhibit Inv expression in the cornified layers. Whilst K14 expression appears stronger in the suprabasal cells of the in vitro HSE than in normal epithelium and some difference in the layers of epithelium is apparent between HEKn-CaY and HEKn, the cells do exhibit the ability to stratify fully to the cornified layer and express all three markers of differentiation in a similar manner. It is important to note that the HSE have thicker cornified layers than normal human skin as it is protected from sloughing which removes much of the cornified layer of normal human skin.

## Discussion

Primary keratinocytes are known to have a limited lifespan in culture. Typically, keratinocytes spontaneously differentiate and quickly reach senescence, becoming unviable for further use in in vitro culture experiments [1]. As such, it is generally suggested that keratinocytes used in investigations of skin biology and wound healing including 3D HSE models be limited to passage 1 to 3 cells [16]. The requirement of repeated isolations of primary keratinocytes can lead to increased variation in experiments and has often lead researchers to rely upon immortalised cell lines, such as the HaCaTs, however these are now recognised to vary greatly in phenotype from primary keratinocytes [9]. Indeed, as we have seen, HaCaTs are unable to fully stratify and form the cornified layer of an epidermis making them unsuitable for more sophisticated in vitro models of skin biology such as the 3D HSE model.

Early work showed the use of feeder layers to support the culture of keratinocytes and that the addition of exogenous agents, such as EGF could greatly increase the lifespan of keratinocytes in culture [18,19]. There is however a push towards culture of keratinocytes for clinical use without the use of a feeder to reduce the dependence upon animal products and also the recognition that keratinocytes in monolayer are more suited to biological investigations than those grown in the presence of a fibroblast feeder layer [20,21]. The Rho kinase inhibitor Y-27632 inhibits terminal differentiation and increases proliferation in keratinocytes in culture [11,13]. The addition of Y-27632 to normal keratinocyte growth media has been shown to maintain proliferation indefinitely provided the keratinocytes are supported by an irradiated fibroblast feeder layer [12]. Whilst Chapman et al indicated the requirement of a fibroblast feeder layer to maintain keratinocytes beyond PD35, we have shown that with the combination of Y-27632 and very low calcium, it is possible to maintain a healthy, responsive population of keratinocytes in monolayer culture beyond this point. Whilst we saw senescence in keratinocytes cultured in very low calcium media alone by PD35, the viability of those we cultured in very low calcium supplemented with Y-27632 remained high beyond 40PD with a continued steady growth rate. Thus, we demonstrate that Y-27632 supplementation in combination with low calcium increases the proliferative capacity, expansion potential and lifespan of primary human keratinocytes without the need for a feeder layer.

We have demonstrated that HEKn-CaY in monolayer culture maintain a basal phenotype with no expression of differentiation makers at the protein or RNA level. Critically, the removal of the Y-27632 from the culture media allows the cells to behave as early passage primary keratinocytes, even after extended serial culture. Identification of agents which stimulate differentiation of keratinocytes in a 2D, monolayer setting is also possible using the immunohistochemical markers of early keratinocyte differentiation K10 compared with basal marker K14 as we have shown induction of differentiation only occurs with the addition of high calcium to the media, not simply immediately after removal of Y-27632. This may be important in screening agents to accelerate the reformation of skin barrier function post injury or in various dermatological conditions. Moreover, the same pool of HEKn-CaY may also be used to set up 3D models of human skin which can then be used in sophisticated *in vitro* assays. Using passage 10 Y-27632 treated keratinocytes and passage 3 keratinocytes cultured in normal low calcium media as a control from the same donor source, we have successfully generated 3D HSE’s, demonstrating the ability of these prolonged lifespan cells to fully stratify and retain their original, primary characteristics. Therefore, many more experiments may be set up from one donor sample, increasing the reproducibility and accuracy of research. Our studies suggest that a combination of very low calcium and the addition of ROCK inhibitor can prolong the lifespan of keratinocytes in the absence of a feeder layer enabling the rapid expansion of basal keratinocytes which retain a normal primary phenotype whilst being capable of full differentiation and can used in a range of in vitro assays of skin biology including monolayer screening and 3D HSE models.
